# An intracranial pressure-derived index monitored simultaneously from two separate sensors in patients with cerebral bleeds: comparison of findings

**DOI:** 10.1186/1475-925X-12-14

**Published:** 2013-02-13

**Authors:** Per Kristian Eide, Wilhelm Sorteberg

**Affiliations:** 1 Department of Neurosurgery, Oslo University Hospital, Rikshospitalet, Oslo, Norway; 2 Faculty of Medicine, University of Oslo, Oslo, Norway

## Abstract

**Background:**

In an attempt to characterize the intracranial pressure-volume compensatory reserve capacity, the correlation coefficient (R) between the ICP wave amplitude (A) and the ICP (P) level (RAP) has been applied in the surveillance of neurosurgical patients. However, as the ICP level may become altered by electrostatic discharges, human factors, technical factors and technology issues related to the ICP sensors, erroneous ICP scores may become revealed to the physician, and also become incorporated into the calculated RAP index. To evaluate the problem with regard to the RAP, we compared simultaneous RAP values from two separate ICP signals in the same patient.

**Materials and Methods:**

We retrieved our recordings in 20 patients with cerebral bleeds wherein the ICP had been recorded simultaneously from two different sensors. Sensor 1 was always a solid sensor while sensor 2 was a solid sensor (Category A), a fluid sensor (Category B), an air-pouch sensor (Category C), or a fibre-optic sensor (Category D). The simultaneous signals were analyzed with automatic identification of the cardiac induced ICP waves, with subsequent determination and comparison of the Pearson correlation coefficient between mean wave amplitude (MWA) and mean ICP (RAP) for 40 6-s time windows every 4-min period.

**Results:**

A total of 23,056 4-min RAP observations were compared. A difference in RAP ≥0.4 between the two signals was seen in 4% of the observations in Category A-, in 44% of observations in Category B -, in 20% of observations in Category C -, and in 28% of observations in Category D patients, respectively. Moreover, the combination of a RAP of <0.6 in one signal and ≥0.6 in the other was seen in >20% of scores in 3/5 Category A -, in 3/5 Category B -, in 5/7 Category C - and 1/3 Category D patients.

**Conclusions:**

Simultaneous monitoring of the ICP-derived index RAP from two separate ICP sensors reveals marked differences in the index values. These differences in RAP may be explained by erroneous scoring of the ICP level. This will hamper the usefulness of RAP as a guide in the management of neurosurgical patients.

## Introduction

Monitoring of the intracranial pressure (ICP) remains a cornerstone in the intensive care management of neurosurgical patients [1-3]. Besides scoring the level of ICP (mean ICP), monitoring of the cardiac-induced ICP waves has lately received attention [4-6]. The management goal is then to keep the mean ICP <20-25 mmHg [1,7], or the mean ICP wave amplitude (MWA) <5 mmHg [5]. In an attempt to characterize the intracranial pressure-volume compensatory reserve capacity some authors have in addition calculated ICP-derived indices [6,8,9]. An index measuring the correlation coefficient (R) between the ICP wave amplitude (A) and the mean ICP (P) level (referred to as RAP) has thus been applied in the surveillance of patient groups such as those suffering from traumatic brain injury (TBI), cerebral bleeds or hydrocephalus [8,10-12]. With an upper normal threshold level of about + 0.6 [3,8,11-14] a RAP approaching +1 has been considered as indicative of reduced compensatory reserve capacity.

A crucial, though yet less recognized, aspect of monitoring the level of ICP is that it may become altered by electrostatic discharges in the hospital environment [15], or through human factors, technical factors and technology issues related to the ICP sensors [16]. The consequence of these changes in baseline pressure is then erroneous ICP revealed to the physician [16]. Using these erroneous ICP values when calculating an index, e.g. the RAP, one could anticipate errors being incorporated into the index as well. To see if this in fact occurs, we designed a study wherein simultaneous RAP values derived from two separate ICP sensors in the same patient were compared. The concept of simultaneous measurements from two separate sensors in the same patient was used because erroneous ICP values usually do not become introduced at the same time in both sensors [16]. To this end, and with special emphasize on differences in RAP between the two signals, ICP scores from 20 patients with cerebral bleeds wherein the ICP had been monitored simultaneously from two separate sensors were analyzed.

## Methods

### Patient recordings

The ICP recordings were retrieved from patients managed for aneurysmal subarachnoid haemorrhage (SAH) and/or intra-cerebral haemorrhage (ICH) at the Department of Neurosurgery, Oslo University Hospital – Rikshospitalet during the time period 2002–2011. All patients wherein management included simultaneous monitoring from two separate ICP sensors were included.

The Regional Committee for Medical and Health Research Ethics (REK) of Health Region South-East, Norway approved the study as a quality study (2010/1328). The study was also approved by the Oslo University Hospital – Rikshospitalet as a quality study (2010/16315).

### ICP monitoring and analysis

The setup for the simultaneous ICP monitoring was as follows: Sensor 1 was always a solid (strain-gauge) sensor (Codman Microsensor, Codman MicroSensor, Johnson and Johnson, Raynham, Massachusetts, USA), while Sensor 2 was either (a) another solid sensor (Codman Microsensor, Codman MicroSensor, Johnson and Johnson, Raynham, Massachusetts, USA; Category A), (b) a fluid sensor (Edward’s fluid sensor) connected to an external ventricular drain (Truwave PX-600F Pressure Monitoring Set, Edwards Life sciences LLC, Irvine, CA, USA; Category B), (c) an air-pouch sensor (Spiegelberg intraparenchymal probe 3PN, Spiegelberg KG, Hamburg, GE; Category C), or a fibre-optic sensor (Camino OLM ICP sensor, Camino Laboratories, San Diego, CA; Category D). Both ICP sensors were implanted at the same time.

The ICP sensors were introduced to the intracranial compartment either via a small burr hole and a minimal opening in the dura or via the craniotomy used for aneurysm clipping/hematoma evacuation. The solid sensor was placed within the brain parenchyma, and connected via cable to the ICP Express (Codman ICP Express, Johnson and Johnson, Raynham, Massachusetts, USA). The fluid sensor was connected outside the patient to an external ventricular drain (EVD) that had been placed in the ventricular fluid, while the air-pouch sensor was placed in the ventricular fluid, and connected to a Spiegelberg ICP Monitor (Spiegelberg KG, Hamburg, Ge). The fibre-optic sensor was placed within the brain parenchyma, and connected via cable to the MPM-1 Camino monitor (Camino Laboratories, San Diego, CA). The ICP signals from all sensors were passed to a vital signs Siemens 9000 XL Series Monitor (Siemens Medical Systems Inc., Danvers, MA, USA). By means of the Siemens Infinity Gateway Software (Siemens Medical Systems Inc., Danvers, MA, USA), the continuous ICP signals were transferred online via the hospital network to a computer server and stored as raw data files (sampling rate 100 Hz).

The analysis of the continuous ICP waveforms was done using a previously published method for automatic identification of cardiac induced ICP waves [17]. The method has been implemented in the software (Sensometrics Software, dPCom As, Oslo, Norway). Each single ICP wave becomes identified by its beginning and ending diastolic minimum pressure, and its systolic maximum pressure, and various single ICP wave parameters are determined, such as the pressure difference between the beginning diastolic and systolic pressures (dP). For 6-s time windows containing a minimum of 4 cardiac induced ICP waves, the mean ICP and the mean ICP wave amplitude (MWA) were determined.

**Figure 1:**
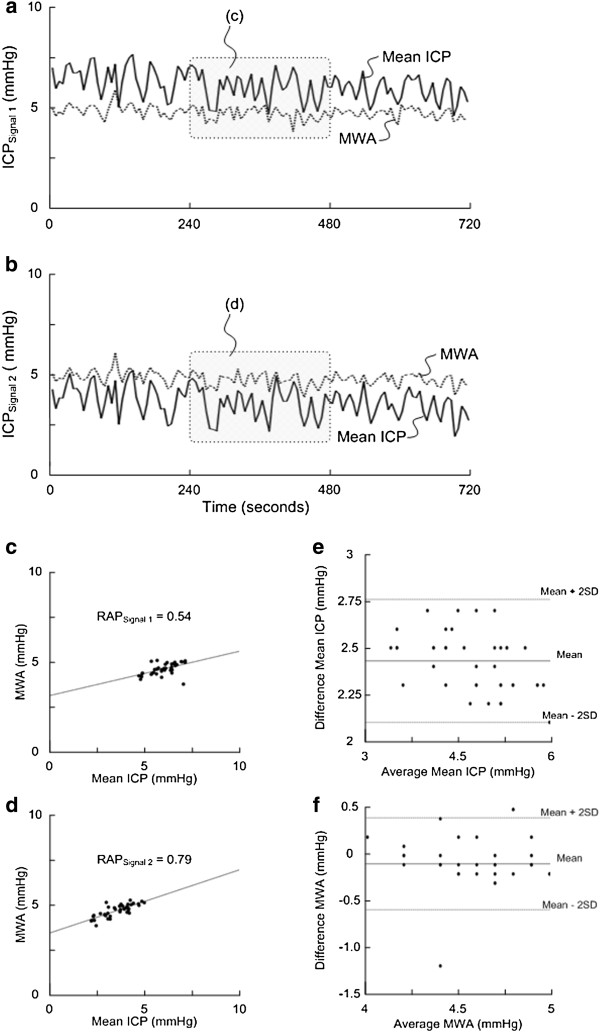
**Illustration of the method of determining the RAP in patient 1.** Following automatic identification of the cardiac induced single intracranial pressure (ICP) waves, the mean ICP and mean wave amplitude (MWA) are determined for every 6-s time window. Trend plots of mean ICP and MWA determined during the same 6-s time windows are shown for Signals 1 (**a**) and 2 (**b**) over a 12 min period (representing three 4-min periods, and 120 6-s time windows). For Signal 1 (**a**) the average (±standard deviation) of mean ICP was 6.1±0.7 mmHg and of MWA 4.7±0.3 mmHg; while for Signal 2 (**b**) mean ICP 3.6±0.7 mmHg and MWA 4.8+0.3 mmHg (mean difference of ICP −2.4±0.2 mmHg; mean difference of MWA 0.09±0.17 mmHg). RAP is determined as the Pearson correlation coefficient between mean ICP and MWA during subsequent 4 min periods (representing 40 6-s time windows). For the 20 patients in this study, we compared the RAP values during the same 4-min periods for Signals 1 and 2. For the 4-min period shown here, RAP was 0.54 for Signal 1 (**c**) while 0.79 for Signal 2 (**d**). The difference in RAP during this 4 min period was related to the difference in mean ICP [mean ICP: 5.9±0.7 mmHg (Signal 1) vs. 3.5±0.8 mmHg (Signal 2)], while the difference in MWA was marginal [MWA: 4.6±0.3 mmHg (Signal 1) vs. 4.7±0.3 mmHg (Signal 2)]. This is further illustrated in a Bland-Altman [33] plot showing less agreement between mean ICP (**e**; mean difference = 2.43 mmHg; mean + 2SD = 2.76 mmHg; mean – 2SD = 2.10 mmHg), while higher agreement between MWA of Signals 1 and 2 (**f**; mean difference = −0.09 mmHg; mean + 2SD = 0.41 mmHg; mean – 2SD = −0.59 mmHg).

**Figure 2:**
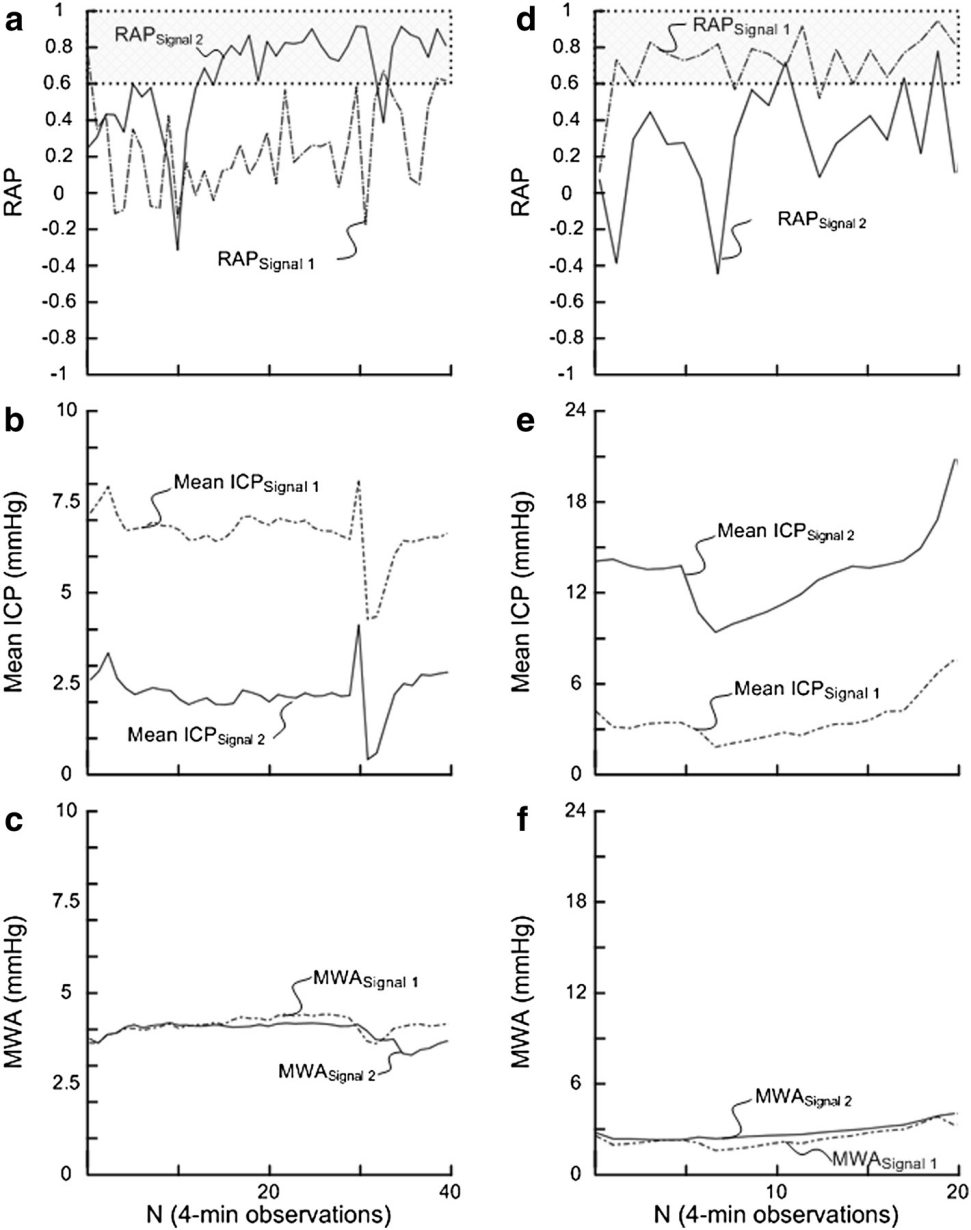
**Trend plots of RAP, mean ICP and MWA of Signals 1 and 2 in patients 1 and 20.** For patient 1 the trend plots of (**a**) RAP determined during consecutive 4-min periods for Signal 1 and 2 show marked differences (average of RAP_Signal 1_ 0.22; average of RAP_Signal 2_ 0.63), accompanied with marked differences in (**b**) mean ICP (average of mean ICP_Signal 1_ 6.6 mmHg; average of mean ICP_Signal 2_ 2.2 mmHg) while minor differences in (**c**) MWA (average of MWA_Signal 1_ 4.0 mmHg; average of MWA_Signal 2_ 3.9 mmHg). Similarly, for patient 20 the trend plots of (**d**) RAP revealed marked differences (average of RAP_Signal 1_ 0.72; average of RAP_Signal 2_ 0.29), and markedly different (**e**) mean ICP (average of mean ICP_Signal 1_ 3.5 mmHg; average of mean ICP_Signal 2_ 13.2 mmHg) despite similar (**f**) MWA (average of MWA_Signal 1_ 2.4 mmHg; average of MWA_Signal 2_ 2.8 mmHg). The horizontal lines at RAP 0.6 illustrate a commonly used upper normal threshold for RAP.

The correlation coefficient (R) between the ICP wave amplitude (A) and ICP (P) level (RAP) was determined during consecutive 4-min time periods [8,14]. Thus, the RAP represents the Pearson correlation coefficient between the MWA and the mean ICP in 40 6-s time window periods. Since we compared RAP of two simultaneous ICP signals, the RAP of Sensors 1 and 2 were derived from simultaneous 6-s time windows (Figure 1a-b). For every consecutive 4-min period the software hence determined the Pearson correlation coefficient (RAP) values of the two ICP signals (Figure 1c-d). The RAP scores could then be trended as shown in Figure 2.

The Pearson correlation coefficient is a measure of the strength of a relationship between two variables and may range from −1 to +1. A negative correlation coefficient occurs when one variable changes in the opposite direction of the other; whereas a positive correlation coefficient indicates that both variables change in the same direction (Figure 1c-d). The closer the correlation coefficient is to +1, the stronger is the relationship between the two variables.

Based on previous findings, the assumptions for using the Pearson correlation coefficient were fulfilled: both the ICP and the MWA are continuous and independent observations that follow a normal distribution. For 4-minute intervals, the correlation coefficient between these two observations reflects a linear relationship.

## Results

### Patient recordings

Table 1 gives information with regard to demography, type of cerebral bleed, and the ICP sensor types and locations for the 20 patients.

**Table 1:** Information about demography, type of bleed and ICP sensor type and location in 20 patients with cerebral bleeds

**PatID**	**Age**	**Gender**	**Type of bleed**	**Sensor type**		**Sensor location**	
				**Signal 1**	**Signal 2**	**Signal 1**	**Signal 2**
**Category A**							
1	66	M	SAH (ACOM)	Solid	Solid	Left frontal lobe	Right frontal lobe
2	76	M	ICH (right parieto-occipital)	_“_	_“_	Left frontal lobe	Left occipital lobe
3	39	F	SAH (ACOM)	_“_	_“_	Right frontal lobe	Right occipital lobe
4	72	F	SAH (left MCA)	_“_	_“_	Left frontal lobe	Left frontal ventricular horn
5	59	F	SAH (left MCA)	_“_	_“_	Right frontal lobe	Right frontal ventricular horn
**Category B**							
6	56	M	SAH (BA)	Solid	Fluid	Right frontal lobe	Right frontal ventricular horn
7	48	M	SAH (left MCA)	_“_	_“_	Right frontal lobe	Right frontal ventricular horn
8	60	M	SAH (ACOM)	_“_	_“_	Right frontal lobe	Right frontal ventricular horn
9	50	F	SAH (right VA)	_“_	_“_	Right frontal lobe	Right frontal ventricular horn
10	55	F	SAH (ACOM)	_“_	_“_	Left frontal lobe	Left frontal ventricular horn
**Category C**							
11	66	M	ICH (right frontal)/IVH	Solid	Air-pouch	Right frontal lobe	Right frontal ventricular horn
12	56	F	SAH (right MCA)	_“_	_“_	Right frontal lobe	Right frontal ventricular horn
13	60	F	SAH (BA/left ICA)	_“_	_“_	Right frontal lobe	Right frontal ventricular horn
14	54	M	SAH (left PCOM)	_“_	_“_	Right frontal lobe	Right frontal ventricular horn
15	67	M	SAH (right PCOM)	_“_	_“_	Left frontal lobe	Left frontal ventricular horn
16	71	M	ICH (cerebellum)	_“_	_“_	Right frontal lobe	Right frontal ventricular horn
17	82	F	ICH (cerebellum)	_“_	_“_	Right frontal lobe	Right frontal ventricular horn
**Category D**							
18	60	F	SAH (right ICA)	Solid	Fibre-optic	Right frontal lobe	Right frontal lobe
19	71	M	SAH (ACOM)	_“_	_“_	Right frontal lobe	Right frontal lobe
20	52	F	SAH (left PCOM)	_“_	_“_	Right frontal lobe	Right frontal lobe

*ACOM*: anterior communicating artery; *BA*: basilar artery; *ICA*: internal carotid artery; *ICH*: Intra-cerebral haemorrhage; *IVH*: intra-ventricular haematoma; *MCA*: middle cerebral artery; *PCOM*: posterior communicating artery; *SAH*: Subarachnoid haemorrhage; *VA*: vertebral artery.

### Simultaneous RAPs of Signal 1 and Signal 2

Figure 1 illustrates how RAP may differ between Signals 1 and 2 due to differences in mean ICP level while MWA being close to identical.

Simultaneous RAP scores from Signal 1 and Signal 2 are presented in Table 2. Table 2, left shows the number of 4-min RAP scores in each patient. For all 20 patients combined, a total of 23,056 4-min RAP scores were analyzed. Table 2, middle presents the RAP scores (mean and ± std) for each patient. Values ≥0.6 (upper limit of normality) are highlighted. Table 2, right shows differences in RAP between Signal 1 and Signal 2 exceeding 0.2, 0.4 and 0.6, respectively. A difference in RAP ≥0.4 between the two signals was seen in 4% of the observations in Category A-, in 44% of observations in Category B -, in 20% of observations in Category C -, and in 28% of observations in Category D patients, respectively D (Table 2).

**Table 2:** Comparison of RAP between Signals 1 and 2

**PatID**	**N (4-min RAP observations)**	**RAP (average + std)**		**Differences in RAP between Signals 1 and 2 (N, %)**		
		**Signal 1**	**Signal 2**	**0.2**	**0.4**	**0.6**
**Category A**						
1	685	0.45±0.4	0.42±0.39	315 (46%)	162 (24%)	45 (7%)
2	1,040	0.37±0.22	0.36±0.22	105 (10%)	6 (1%)	1
3	414	0.83±0.17	0.78±0.21	52 (13%)	18 (4%)	7 (2%)
4	604	0.58±0.29	0.65±0.28	115 (19%)	23 (4%)	8 (1%)
5	1,065	0.57±0.38	0.59±0.36	419 (39%)	206 (19%)	78 (7%)
**Category B**						
6	211	0.55±0.39	−0.74±0.20	207 (98%)	201 (95%)	190 (90%)
7	1,499	0.56±0.35	0.11±0.58	1,060 (71%)	838 (56%)	673 (45%)
8	274	0.67±0.37	0.28±0.67	140 (51%)	95 (35%)	70 (26%)
9	191	0.09±0.43	0.13±0.43	130 (68%)	84 (44%)	44 (23%)
10	302	0.88±0.13	0.86±0.14	61 (20%)	17 (6%)	1
**Category C**						
11	1,834	0.62±0.27	0.80±0.17	844 (46%)	347 (19%)	129 (7%)
12	4.777	0.15±0.29	0.16±0.28	2,661 (56%)	1,135 (24%)	427 (9%)
13	1,861	0.33±0.35	0.35±0.32	922 (50%)	345 (19%)	103 (6%)
14	2,361	0.34±0.31	0.30±0.35	1,259 (53%)	573 (24%)	234 (10%)
15	2,022	0.50±2.9	0.38±0.32	923 (46%)	500 (25%)	318 (16%)
16	15	0.31±0.34	0.45±0.40	4 (26.7%)	3 (20%)	2 (13%)
17	764	0.71±0.27	0.69±0.34	197 (26%)	99 (13%)	62 (8%)
**Category D**						
18	1,841	0.66±0.28	0.61±0.31	354 (19%)	114 (6%)	35 (2%)
19	578	0.04±0.36	0.06±0.34	319 (55%)	162 (28%)	87 (15%)
20	718	0.65±0.35	0.48±0.43	339 (47%)	215 (30%)	135 (19%)

*RAP*: Relationship Amplitude – Pressure.

**Figure 3:**
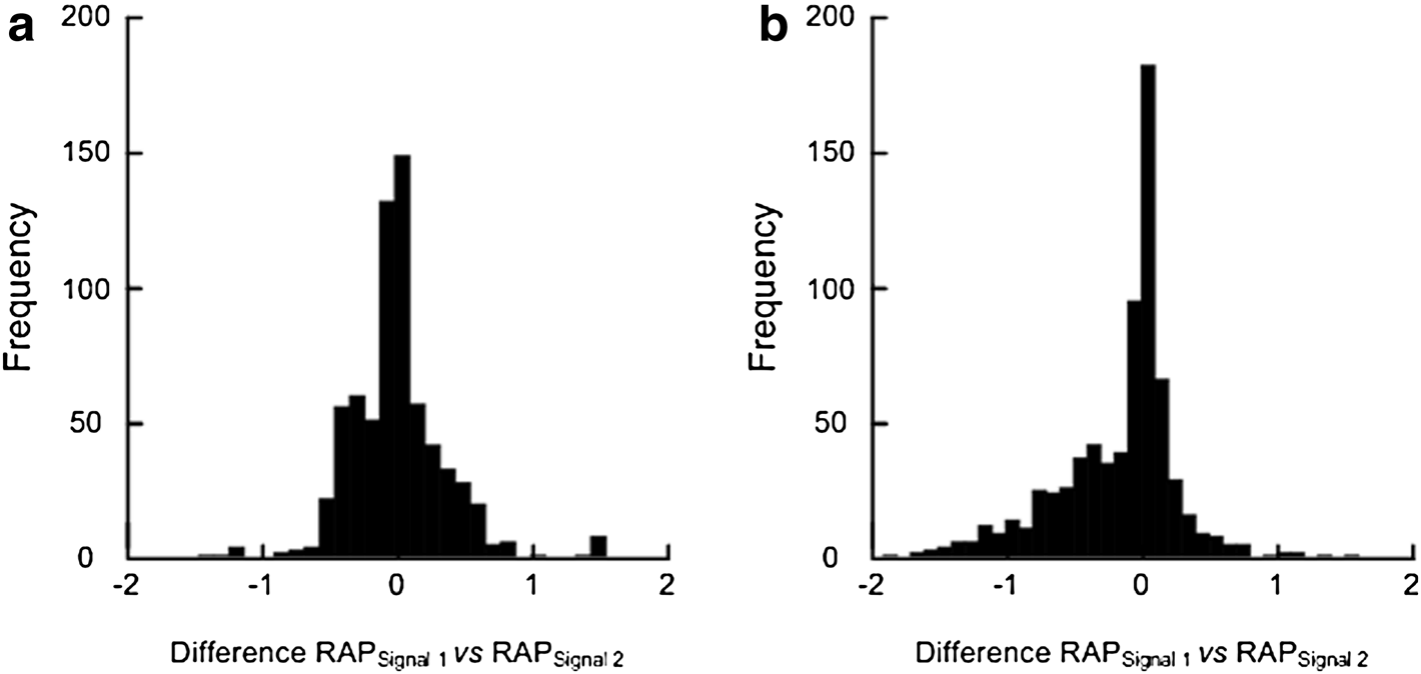
**Histograms show frequency of differences in RAP between identical Signals 1 and 2 in patients 1 and 20.** Histograms of differences in RAP between identical 4-min periods of Signal 1 and 2 are shown for PatID’s 1 [a; mean difference (+std) = 0.02±0.36, n = 685], and 20 [a; mean difference (+std) = −0.17±0.45; n = 718]. The histograms illustrate that despite minor mean difference in RAP between Signals 1 and 2 of all 4-min periods, the frequency of major negative and positive differences in RAP can be extensive.

Figure 2 illustrates how the RAP could differ between Signals 1 and 2. The figure shows short trends of RAP signals in Category A- (Figure 2a), B- (Figure 2b), C- (Figure 2c) and D patients (Figure 2d), respectively. This aspect is further illustrated in Figure 3, showing histograms of the differences in RAP between the two signals for all scores. Examples are presented of patients in Category A (Figure 3a), B (Figure 3b), C (Figure 3c) and D (Figure 3d), respectively.

Table 3 presents the portion of 4-min observations wherein RAP was <0.6 in both signal (left), ≥0.6 in both signals (middle) and <0.6 in one signal and ≥0.6 in the other (right). The combination of a RAP of <0.6 in one signal and ≥0.6 in the other was seen in >20% of scores in 3/5 Category A patients, in 3/5 Category B patients, in 5/7 Category C patients and 1/3 Category D patients, respectively.

**Table 3:** Proportion of 4-min observations with RAP <0.6 in both signals (left), RAP ≥0.6 in both signals (middle) and RAP ≥0.6 in one signal while RAP <0.6 in another signal (right)

**PatID**	**RAP**_**SIGNAL 1**_**<0.6/ RAP**_**SIGNAL 2**_**<0.6**	**RAP**_**SIGNAL 1**_**≥0.6/ RAP**_**SIGNAL 2**_**≥0.6**	**RAP**_**SIGNAL 1**_**≥0.6/RAP**_**SIGNAL 2**_**<0.6 or RAP**_**SIGNAL 1**_**<0.6/RAP**_**SIGNAL 2**_**≥0.6**
**Category A**			
1	308 (45%)	233 (34%)	**144 (21%)**
2	842 (81%)	115 (11%)	83 (8%)
3	21 (5%)	339 (82%)	54 (13%)
4	176 (29%)	319 (53%)	**109 (18%)**
5	319 (30%)	501 (47%)	**245 (23%)**
**Category B**			
6	95 (45%)	0	**116 (55%)**
7	536 (36%)	272 (18%)	**691 (46%)**
8	65 (24%)	118 (43%)	**91 (33%)**
9	156 (82%)	4 (2%)	31 (16%)
10	5 (2%)	270 (89%)	27 (9%)
**Category C**			
11	140 (8%)	1066 (58%)	**628 (34%)**
12	4,306 (90%)	34 (1%)	437 (9%)
13	1,176 (63%)	266 (14%)	**419 (23%)**
14	1,542 (65%)	318 (14%)	**501 (21%)**
15	985 (49%)	390 (19%)	**647 (32%)**
16	10 (67%)	3 (20%)	2 (13%)
17	116 (15%)	494 (65%)	**154 (20%)**
**Category D**			
18	443 (24%)	1,106 (60%)	292 (16%)
19	523 (91%)	10 (2%)	45 (8%)
20	177 (25%)	313 (44%)	**228 (32%)**

*RAP*: Relationship Amplitude – Pressure.

## Discussion

The main finding of the present study was that simultaneous monitoring of the ICP-derived index RAP from two separate ICP sensors in the same patient revealed marked differences in index values. In a setting where erroneous ICP values are introduced at separate points of time in the two sensors, this finding could be anticipated. To our knowledge, this is further the first study wherein RAP values from simultaneous ICP signals are compared.

### ICP derived indices

The rational for introducing ICP-derived indices have been to enhance the diagnostic information of ICP monitoring [3,8]. The most commonly used such index, the correlation coefficient (R) between the ICP wave amplitude (A) and ICP (P) level (RAP), which is considered to be an indicator of the intracranial pressure volume compensatory reserve capacity [3,6,10,14]. The RAP should thus be of particular value in the surveillance of patients with TBI [8,12,14,18], cerebral bleeds and hydrocephalus [10,11,13]. With an upper normal threshold level of about + 0.6 [3,8,11-14], however, the clinical usefulness of this index remains to be determined [4,19,20]. One reason for this could be the erroneous ICP values being incorporated into the index value.

The RAP was originally determined from amplitudes computed using the frequency domain method [8,14]. In contrast, the MWA was presently determined using the time domain method [17]. Although the two methods are not equivalent with regard to computation of ICP wave amplitudes [21], how amplitudes are computed should not affect the results since amplitudes are relative values not being affected by baseline pressure. Also, the concept of RAP being an indicator of intracranial pressure reserve capacity should not be affected by the method of computing amplitudes.

### Measurements of differences in mean ICP between two ICP sensors

We have previously observed markedly different mean ICP levels from two simultaneous ICP measurements [16,22,23]. As we recently reported in the very same ICP recordings as presented here, the differences in mean ICP occurred despite of close to identical MWAs (Patients 1–17; ref [16]; Patients 18–20, ref [22]). The fluctuations in the mean ICP were caused by spontaneous shifts and drifts of the ICP baseline pressure. As the ICP is a pressure value relative to the atmospheric pressure (representing the baseline or reference pressure), any change in baseline pressure obviously will affect the ICP level being scored.

There are three major factors related to hospital environment that may affect the ICP baseline pressure: Human factors, technical issues and technology issues. The most important human factor is erroneous zeroing of the ICP sensor, and mal-positioning of the sensor/catheter. Technical issues include sensor damage that may occur during implantation or at any point during monitoring and sensitivity to electrostatic discharges (ESD’s) [15]. Moreover, when using a fluid system, loss of fluid continuity due to air bubbles and/or partial/total occlusion of the fluid catheter by blood cloths or brain tissue may cause erroneous pressure reproduction. Technology issues relate to the properties of the ICP sensor itself. For the ICP sensors used here, we have shown how the transfer function varies between solid and fluid/air pouch sensors, making the air pouch sensor less useful for reproduction of pressure waveforms [16]. All factors mentioned above may thus contribute to the observed differences in RAP.

### Comparison of RAP scores from two simultaneous ICP signals

In a significant proportion of our observations, there were marked differences in RAP between Sensors 1 and 2 (Table 2). A difference in RAP ≥0.4 between the two signals was hence seen in >20% of the observations in more than half of the patients. It might be questioned whether these differences are of clinical importance. To explore this further, we therefore determined the proportion of 4-min observations wherein the RAP was ≥0.6 in one signal while <0.6 in another (above normal threshold level in one sensor and below in the other). Doing so, we found this setting in >20% of scores in 12/20 patients; moreover, it occurred with every type of sensor being used in the study. This observation is important because in the clinical setting, surveillance of patients is based on the actual monitoring values that are revealed to the observer (physician or nurse).

Due to erroneous ICP scores being incorporated into the RAP scores, its usefulness as a guide in the management of neurosurgical patients will be hampered. In contrast, as the ICP wave amplitude does not become affected by baseline pressure changes, the ICP wave amplitude is a robust parameter. Thus, in a recent study comparing the ICP waveforms ICP wave amplitude, ICP wave slope, and RAP as measures of intracranial compliance in head injury patients, the ICP wave amplitude showed best performance [19].

### ICP sensors

The ICP sensors referred to in this study have been widely used for years; they thus all represent state-of-the art technology for ICP monitoring. While the Edward’s fluid sensor is extensively used for monitoring of fluid-pressures in general, including arterial blood pressure, intraventricular pressure and central venous pressure, dedicated ICP sensors were represented by the solid Codman ICP sensor [24-28], the air-pouch Spiegelberg ICP sensor [29,30], and the fibreoptic Camino ICP sensor [31,32].

## Conclusions

Simultaneous monitoring of the ICP-derived index RAP from two separate ICP sensors reveals marked differences in the index values. The differences in RAP may be explained by erroneous scoring of the ICP level. This will hamper the usefulness of RAP as a guide in the management of neurosurgical patients.

## Abbreviations

ESD: Electrostatic discharge; ICP: Intracranial pressure; MWA: Mean ICP wave amplitude; RAP: Correlation coefficient (R) between ICP wave amplitude (A) and ICP level (P); SW: Single wave; TBI: Traumatic brain injury.

## Competing interests

WSO reports no conflicts of interest. PKE has financial interest in the software company (dPCom A/S) that manufactures the software (Sensometrics® Software), which was used for digital recording of the continuous pressure signals in this study.

## Authors’ contributions

Both authors contributed to conception and design, acquisition and interpretation of data. PKE contributed the bulk of the drafting of the manuscript and WSO contributed with thorough editing of the manuscript. Both authors have read and approved the final manuscript.
